# Steinach and Young, Discoverers of the Effects of Estrogen on Male Sexual Behavior and the “Male Brain”^1,2^

**DOI:** 10.1523/ENEURO.0058-15.2015

**Published:** 2015-11-17

**Authors:** Per Södersten

**Affiliations:** Section of Applied Neuroendocrinology, Karolinska Institutet, S-141 04 Huddinge, Sweden

**Keywords:** Estrogen, history, male brain, sex behavior, sex differences

## Abstract

In the 1930s, Eugen Steinach’s group found that estradiol induces lordosis in castrated rats and reduces the threshold dose of testosterone that is necessary for the induction of ejaculation, and that estradiol-treated intact rats display lordosis as well as mounting and ejaculation. The bisexual, estrogen-sensitive male had been demonstrated. Another major, albeit contrasting, discovery was made in the 1950s, when William Young’s group reported that male guinea pigs and prenatally testosterone-treated female guinea pigs are relatively insensitive to estrogen when tested for lordosis as adults. Reduced estrogen sensitivity was part of the new concept of organization of the neural tissues mediating the sexual behavior of females into tissues similar to those of males. The importance of neural organization by early androgen stimulation was realized immediately and led to the discovery of a variety of sex differences in the brains of adult animals. By contrast, the importance of the metabolism of testosterone into estrogen in the male was recognized only after a delay. While the finding that males are sensitive to estrogen was based on Bernhard Zondek’s discovery in 1934 that testosterone is metabolized into estrogen in males, the finding that males are insensitive to estrogen was based on the hypothesis that testosterone–male sexual behavior is the typical relationship in the male. It is suggested that this difference in theoretical framework explains the discrepancies in some of the reported results.

## Significance Statement

In 1936, the importance of estrogen in male sexual behavior was discovered. This finding went unnoticed when the effect of estrogen in the male was rediscovered in the early 1970s; the original report of the effect of estrogen in the male in 1936 was found only in 2012. An equally significant discovery was made in 1959, when it was found that prenatal treatment with testosterone organizes the brain of a female into a male brain and permanently decreases behavioral estrogen sensitivity. Males cannot be both sensitive and insensitive to estrogens, and this inconsistency may have contributed to the long latency before the significance of estrogen in the male was recognized.

## Introduction

The hypothesis that estrogen, formed in the brain from testosterone in the circulation, is important for sexual behavior in male rats is often dated to 1970 (McDonald et al., 1970). However, the idea was launched 34 years earlier in a report by Steinach et al. (1936; translated in Södersten et al., 2014), which was not mentioned when the effect of estrogen was rediscovered in the early 1970s (Södersten, 2012). Thus, the original article (Steinach et al., 1936), which demonstrated that a behaviorally ineffective dose of estradiol benzoate (EB) synergizes with a likewise behaviorally ineffective dose of testosterone in restoring ejaculation in castrated rats hibernated for a long time, although it was reviewed in detail in 1938 when Steinach was nominated for the seventh time for the Nobel Prize (Liljestrand, 1938). In evaluating Steinach’s work, including the finding that EB stimulates female sexual behavior in male rats, the Nobel Prize Committee acknowledged the behavioral bipotential of the sexes as one of Steinach’s major contributions (Liljestrand, 1938).

Steinach’s hypothesis that the behavioral sex of an animal is reversible by treatment with the hormones of the opposite sex was challenged in the 1950s, when it was discovered that the behavioral sensitivities to gonadal hormones are unequally distributed both within and between the sexes (Grunt and Young, 1952; Phoenix et al., 1959). Most important, these authors demonstrated that the sensitivities to gonadal hormones of adult animals are determined by exposure to testosterone in early development. This discovery stimulated the search for sex differences in the brain, which are causally related to sex differences in sexual behavior. This was a major step ahead; the field flourished by focusing on the following key concepts: hormone specificity, tissue sensitivity, activation and organization of the tissues mediating mating behavior, and the associated idea that males are relatively insensitive to estrogen (Phoenix et al., 1959; Young, 1961).

It is suggested that the emergence of these ideas is why the significance of Steinach’s work was not recognized immediately by behavioral neuroendocrinologists.

## Discovery of the Effects of Estrogen in Male Rats

When, in the 1930s, treatment with synthetic gonadal hormones replaced transplantation of the gonads, the method that Steinach had used to demonstrate sex reversal of reproductive behavior and anatomy, his group published two studies on the effects of estrogen in male rats.

### Lordosis in castrated rats, and lordosis and ejaculation in intact rats

In the first study, Kun (1934) castrated rats at 4-6 months of age, and 3-11 months later the rats were injected once with EB, and tested with males 48 h later. With 2.9 μg of EB, only one of four rats showed lordosis, but with ≥5.8 μg of EB, all rats responded. Intact, sexually active rats injected with a higher dose, 23.2 μg of EB, also showed lordosis when tested with males and continued ejaculating when tested with females; their bisexual behavior was highlighted in the title of the article.

Although the behavior was reported as all-or-none rather than quantitatively in these experiments, it should be recognized that, at the time, many scientists questioned whether behavior can be measured at all and even if it is possible to use a mathematical model in biology (Beach, 1981; Södersten, 2012). Despite these constraints, the methods permitted the demonstration that the dose of EB necessary for the induction of lordosis in intact rats is higher than the dose needed in castrated rats, a finding that has been replicated using modern methods (Butler et al., 2001).

A note on hormone doses is appropriate at the beginning of this overview. The ovarian hormone that produces estrus was referred to as Folliculin, estrin, Progynon, or theelin (theelico is the Greek for female). Schering AG launched 17β EB, Progynon, in 1928. One “dragée” contained 250 ME (Mäuseeinheit) estradiol [1,3,5(10)-estratrien- 3,17β-diol]. The ME (Mouse Unit) was subsequently replaced by IE [Internationale Einheit (International Unit [IU])]. Because the effect of the estrogen preparations varied depending on their purity and the maintenance conditions of the animals, the doses used in the different experiments are not necessarily comparable. Progynon B, 1 mg of 17β EB (50,000–80.000 IU)/ml oil was launched in 1932. This is the EB commonly used in behavioral research.

### Potentiation of testosterone-induced ejaculation in castrated rats

In the second study, Steinach et al. (1936; Södersten et al., 2014) first showed that injection of EB, but not androgens, replicated the effect of testicular extracts on cerebral blood flow, an assay of an effect on the brain. It was then hypothesized that estrogen also acts on the brain to stimulate sexual behavior by synergizing with androgens, as had been demonstrated in the seminal vesicles (Freud, 1933). The hypothesis was verified; the threshold dose of testosterone necessary for the induction of ejaculation in castrated rats was reduced 10-fold by the addition of EB. Given alone, these doses of EB or testosterone had no effect. A third experiment showed that males convert androgens into estrogens, confirming previous reports by Zondek (1934a,b; Zondek and von Euler, 1934).

Thus, in 1936 Steinach documented the role of estrogen in the sexual behavior of male rats.

Although Steinach’s discoveries were recognized in other fields, they have been overlooked in behavioral neuroendocrinology. Beach (1948) noted that androgens are converted into estrogens, but he did not mention that Zondek had launched this hypothesis, and left the synergistic effect of estradiol and testosterone on ejaculation without notice.

The development of quantitative methods in behavioral neuroendocrinology by Young and Beach led to the important discovery of individual and sex differences in responses to gonadal hormones, and shifted the focus from the sex similarities that Steinach had studied to sex differences (Feder, 1981).

## The Estrogen-Insensitive Male

Today, the demonstration that estrogen mediates the effect of testosterone on sexual behavior in the male is considered “one of the most important discoveries of late twentieth century neuroendocrinology” (Ball and Balthazart, 2012). Steinach had already made this discovery in 1936. However, before realizing the significance of this idea, the neuroendocrinologists of sexual behavior examined the relationships among the sex of the animal, the gonadal hormone, and the display of sexual behavior.

### “The problem of hormone specificity”

First of all, the concept of “tissue sensitivity” was introduced to account for the finding that individual differences in the display of sexual behavior by male guinea pigs cannot be overcome by the administration of large amounts of testosterone (Grunt and Young, 1952). Second, Beach (1948) had reviewed the evidence for the eight possible combinations among sex, gonadal hormone, and sexual behavior, and out of these combinations Young (1961) considered the “male sex–testosterone–masculine behavior” and the “female sex–estrogen–feminine behavior” relationships to be “typical.” Interestingly, while he regarded the “estrogen–masculine behavior” a “common relationship” in the female, he did not think that was a strong relationship in the male. Hence, the tissues of males were thought to be sensitive to testosterone and less sensitive to estrogen. Third, at the time, testosterone was considered to be the “male” hormone, and estrogen was considered to be the “female” hormone (Ball and Balthazart, 2012). On this background, the “problem of hormone specificity” was addressed in a study on male guinea pigs.

In that study, guinea pigs were castrated, and, beginning 8 d later, they were injected with estrogens or testosterone for 16 weeks and tested weekly for sexual behavior (Antliff and Young, 1956). Two castrated guinea pigs were injected with testosterone propionate (TP), nine were treated with estrone, five were treated with 17α EB (rather than 17β EB), and one guinea pig served as an untreated control. In order to make up for the differences in the number of animals in these groups, the data from 10 intact and 5 castrated males from older experiments were used, but no addition to the 2 TP-treated males was made.

While no statistical analysis was undertaken, it is interesting that the nine estrone-treated guinea pigs maintained an average score of sexual behavior over the first 10 weeks of the experiment that was only slightly lower than the sex score of the intact guinea pigs and was similar to the score of the two TP-treated guinea pigs. Although they failed to ejaculate over the subsequent 6 weeks, eight of the estrone-treated guinea pigs continued mounting with relatively high frequencies.

Previously, Beach and Holz (1946) had reported that if male rats were castrated on the day of birth, they failed to ejaculate when treated with TP as adults because their penis has not developed normally. As a consequence, the display of mounts without intromission increases. The behavior of these rats was similar to that of the estrone-treated castrated guinea pigs (Antliff and Young, 1956), and so estrone might have failed to maintain the morphology of the penis in guinea pigs after castration, just as EB fails to stimulate penile morphology in castrated rats (Södersten, 1973).

By contrast, 17α EB did not help guinea pigs maintain sexual behavior after castration. Although it was known already that 17α EB is a very weak estrogen (Perlman et al., 1955), the notion that estradiol does not stimulate sexual behavior was supported by a subsequent study in which 17β EB, rather than 17α EB, was ineffective in stimulating sexual behavior in castrated guinea pigs (Alsum and Goy, 1974). Young (1961) used the results on estrone and 17α EB to support the notion that male guinea pigs are insensitive to estrogen, but estrone was clearly effective in maintaining sexual behavior after castration; it remains unknown why estradiol was not.

Thus, by the end of the 1950s, the male was thought to be insensitive to estrogen. By introducing the idea of hormonal organization of the tissues mediating mating behavior, Phoenix et al. (1959) offered a powerful explanation for individual differences as well as sex differences in the behavioral sensitivities to gonadal hormones as they were understood at the time.

## The Era of Activation and Organization

Relying on prevailing concepts of the hormonal organization of the genital tract (Dantchakoff, 1949) and the ovulatory surge of luteinizing hormone secretion (Everett et al., 1949; Harris, 1955), Phoenix et al. (1959) suggested a similar “organizing” effect of testosterone of the “neural tissues mediating mating behavior.” While the activation of sexual behavior by gonadal hormones in gonadectomized adult animals had been demonstrated to be temporary and reversible, the organization of neural tissues was hypothesized to be permanent. The suggestion was logical, timely, and compelling.

In the introduction to their article, Phoenix et al. (1959) pointed out that gonadal hormones “bring to expression the patterns of behavior previously organized …” , thus formalizing the activation–organization dichotomy. Beach (1947) had used these concepts already, and on both accounts, the notion of activation is clear cut. By contrast, the concept of organization is complex. Phoenix et al. (1959) discussed three possibilities.

First, several studies had suggested that genes and experience have “an *organizing action* on the development of the copulatory behavior” (italics added; Valenstein et al., 1955; Goy and Young, 1956; Zimbardo, 1958; but see Beach, 1942). The ways in which organization takes place in this manner were not considered.

Second, the hypothesis that “… hormones have an *organizing action* in the sense of patterning the responses an individual gives to such substances” (italics added) was considered “long rejected.” Organization in this sense is somewhat unclear but is similar to Steinach’s idea that in both sexes the presence of the gonad of the opposite sex leads to “psychosexual transformation” (psychosexuelle Wandlung) of the sexual behavior into that of the opposite sex (Södersten, 2012).

Third was “the possibility that androgens or estrogens reaching animals during the prenatal period might have an *organizing action* that would be reflected by the character of adult sexual behavior” (italics added). This is the key concept of organization. In support, Phoenix et al. (1959) pointed out that Dantchakoff (1938a,b,c) had found that female guinea pigs born to mothers treated with TP developed male sexual organs and showed male sexual behavior as intact adults. Furthermore, Young’s group had reported that perinatal treatment with TP reduces behavioral sensitivity to EB and progesterone (P), and affects uterine morphology in adult female rats (Wilson et al., 1940). Beach (1981) remarked that the evidence presented in that study “was sufficient for formulation of the ‘organization theory’ of hormonal action on the developing brain, but the point was somehow missed, only to be re-discovered 19 years later.”

Fifty years on, Phoenix (2009) wrote: “…what was new [in the 1959 study] was very new… the concept that … the brain had been masculinized.” The “male brain” thus suggested is used in the following review of the article by Phoenix et al. (1959) rather than the lengthy neural tissues mediating mating behavior. Surprisingly, this article, which has been very influential, has never been examined in detail.

## Review of Phoenix et al. (1959)

### Literature review

The new idea that the sex is in the brain rather than in the hormone, relied not only on the new ideas of hormone specificity and tissue sensitivities, but also on a reconsideration of the literature.

Although Kun (1934) had shown that EB-treated male rats display lordosis, it was now suggested that they do not: “Ball (1937) demonstrated that female hormones, instead of feminizing the castrated male rat, as Kun had reported, increased their male activity.” However, the results of the study by Ball (1937) were suggestive rather than conclusive, and they actually supported what Steinach had reported.

In her first experiment, Ball (1937) injected three castrated male rats with estrin, and subsequently with increasing doses of EB. One of the rats ejaculated, but only after the treatment, and the other two rats showed no sexual activity. Three new castrates similarly treated with EB also showed only little male behavior. Ball (1937) pointed out that her study on mounting and ejaculation could not be compared with the study by Kun (1934) on lordosis, and went on to study lordosis as well as mounting and ejaculation in both male and female rats (Ball, 1939). Six gonadectomized males and three females were implanted with a pellet containing theelin (Veler et al., 1930). Without reporting her data, Ball (1939) observed a low level of female sexual behavior in the females, but not in the males. The subsequent injection of increasing doses of EB had no effect in the females, but stimulated a “very low level of female behavior in the males.” Addition of P “may have hastened the appearance of lordosis” in the males but “failed to have the slightest effect on their castrated sisters.”

Aware of the limitations of her studies, Ball (1939) concluded the following: “… that estrogen is capable of producing female sex behavior in animals born males … lordosis was definite, vigorous and repeated four or five times in any single test and every animal showed it for at least one day,” a fact that “*merely confirms what Steinach has claimed for many years*” (italics added).

In addition, four of six castrated EB-treated rats and three of six intact male rats “copulated repeatedly” with receptive females when tested, although ejaculation did not occur and intromission was “uncertain.” However, Ball (1939) pointed out that in her previous experiment, EB had induced ejaculation, and concluded that “castrated males copulate also like males when given the female hormone.”

Phoenix et al. (1959) were right in that the results of the study by Ball (1939) suggested that estrogen stimulates mounting and ejaculation in male rats. In subsequently reviewing the same data, Young (1961) concluded that “estrogenic substances were not strongly effective in stimulating masculine behavior” because he focused on the experiments performed on guinea pigs. However, neither Phoenix et al. (1959) nor Young (1961) evaluated the evidence on lordosis correctly, and they did not comment on the display of bisexual behavior by EB-treated intact rats (Kun, 1934) or on the synergistic effect of EB and testosterone on sexual behavior in castrated rats (Steinach et al., 1936).

### Methods

To test the “organization hypothesis,” one group of guinea pigs was injected with 10–20 mg of TP, and another group was injected with 49–63 mg of TP during various periods of pregnancy. The mothers injected with the lower dose of TP gave birth to “unmodified females” (i.e., females with unchanged external genitalia). By contrast, the mothers injected with the higher dose of TP gave birth to “hermaphrodites” (i.e., females with “external genitalia indistinguishable from those of males,” which will be referred to as a penis in this review). The unmodified females were critical for testing the hypothesis that the brain, rather than peripheral tissues, had been organized into a male brain.

The animals were gonadectomized, but not at the same time, and when they were adult, an unequal number of animals from these groups were treated with 1.66, 3.32, or 6.64 μg of EB followed by 0.2 mg of P 36 h later and were tested for lordosis over 12 h by manually stimulating the flank-perineum area (Young et al., 1937). The animals were also tested for mounting before and after hormone treatment, but the method was not described. To test whether the effects were permanent, the EB plus P treatment and behavioral testing were repeated twice but only with some of the animals; the males were not retested.

A note on the unusual doses of EB seems appropriate. At the time, batches of 17β EB for injections were marked in IU per milliliter, and, because 10,000 IU of EB equals 166 μg, Phoenix et al. (1959) probably diluted these in the easiest manner to obtain doses of 1.66, 3.32, and 6.64 μg of EB.

Five hermaphrodites, five control females, and eight untreated males were gonadectomized, but not at the same time, and were injected with TP over 16 d and tested for mounting behavior as adults. There were no unmodified females in this experiment, and the animals were not retested.

### Results

The responses to 3.32 μg of EB plus P will be considered because there was no relationship between the dose of EB and the display of lordosis or mounting, and this was the only dose used in the retests. The results in test 1 and test 3 will be considered, because the results in test 2 and test 3 were similar.

#### Effect of EB plus P on lordosis

In test 1, very few hermaphrodites and males showed lordosis, but most unmodified and all control females did. Probably for this reason, only three hermaphrodites and no males were retested, and the hermaphrodites did not show lordosis in test 3. The display of lordosis by the hermaphrodites and the males is, therefore, not considered further in this context.

**Figure 1.**
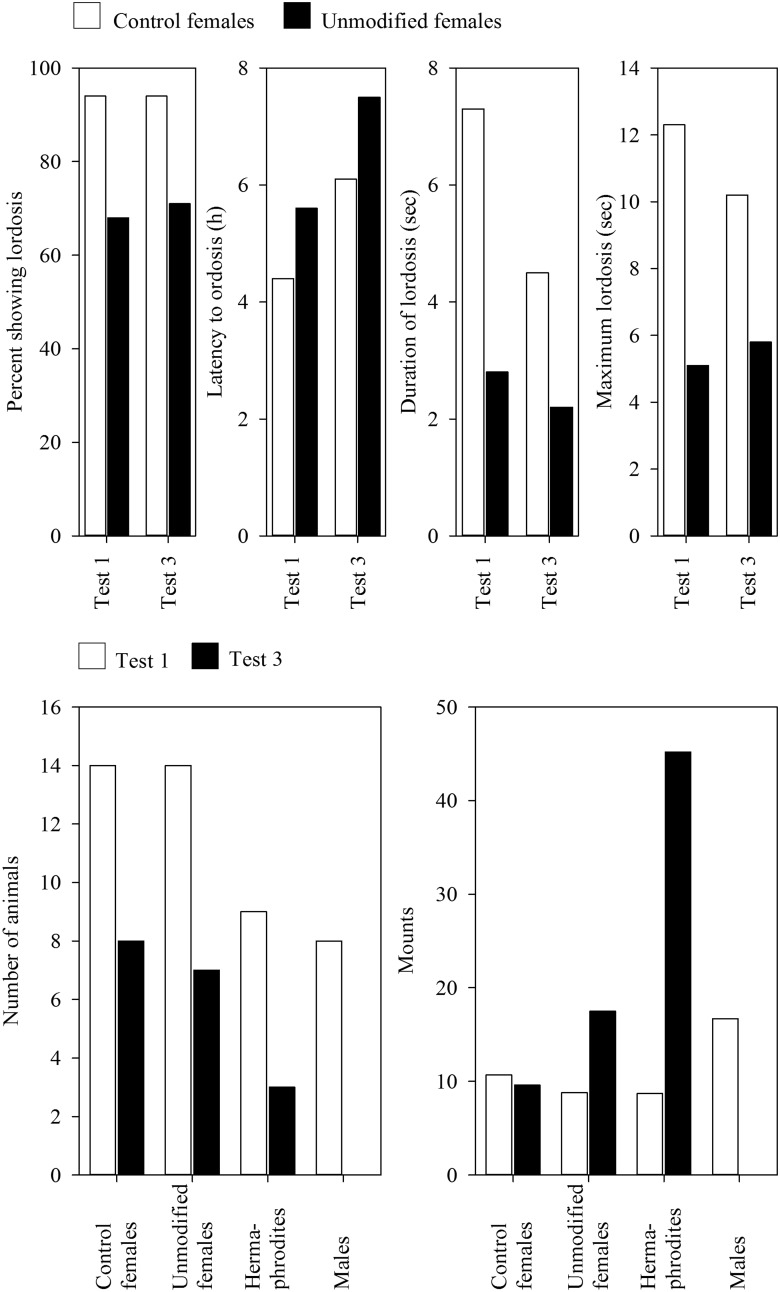
Measures of lordosis (top) and number of animals and mounting (bottom) in female and male guinea pigs treated with 3.32 μg of estradiol benzoate and 0.2 mg of progesterone and tested three times, with 3–5 months between tests (the results are from test 1 and test 3). The animals were born to untreated mothers (control females and males) or to mothers treated with testosterone propionate in doses that produced female offspring with unmodified external genitalia (unmodified females) and females with external genitalia macroscopically indistinguishable from a penis (hermaphrodites). No measures of variability were reported in the original article. Figure redrawn from Phoenix et al. (1959) with permission.

The main results were obtained in the 14 control females and 14 unmodified females in test 1, and in the 8 control females and the 7 unmodified animals in test 3 (Fig. 1). While there was no significant difference in the number of animals showing lordosis, and only a minor difference in the latency to lordosis, the duration of lordosis and the maximum lordosis were shorter among the unmodified females than among controls in test 1 (Fig. 1). Rather than undertaking a between-group comparison in test 3, the authors made within-group comparisons, and the only statistically significant effect was a decrease in the duration of lordosis among the control females (Fig. 1). However, comparisons between the control females and the unmodified females suggest that the between-group differences were smaller in test 3 than in test 1 (Fig. 1).

#### Effect of EB plus P on mounting

The differences in mounting among the experimental groups were conspicuous, and they are shown in relationship to the number of animals in test 1 and test 3 in Figure 1.

First of all, the hermaphrodites and the males mounted without hormone treatment, but the control females and the unmodified females did not. Conversely, treatment with EB plus P had no effect on the hermaphrodites and the males, but stimulated mounting among the unmodified and control females; therefore, these groups did not differ in mount frequency in test 1. Note, however, that the males mounted more than all other groups in test 1 (Fig. 1).

While the frequency of mounts was similar in test 1 and test 3 among controls, the unmodified females mounted twice as much in test 3 compared with test 1, a statistically significant within-group increase in mounting, and the three hermaphrodites mounted eight times more in test 3 compared with test 1 (Fig. 1). However, because they retested so few of these animals and used within-group comparisons, the authors noted that “the increase could not be evaluated statistically.” These marked differences between the prenatally TP-treated animals and the controls indicate that mounting had increased in these groups. How this effect relates to any sex difference is impossible to determine because there were no males in test 3 (Fig. 1). However, the data should be cautiously interpreted because of the variable number of animals that were tested and retested (Fig. 1).

#### Effect of TP on mounting

The hermaphrodites and the males mounted much more than the control females after treatment with TP. There were no unmodified females in this experiment, and the animals were not retested.

## Discussion

The discussion was essentially conceptual, aiming at extracting the “neural tissues” from the “tissues mediating mating behavior.”

### Lordosis and the penis

The conclusion that prenatal treatment with TP suppresses the capacity of female guinea pigs for showing lordosis in response to treatment with EB plus P in adulthood was clearly supported by the results from the hermaphrodites and the males, but less clearly by the results from the unmodified females. In fact, the unmodified females were not compared with the controls in the final test, which was important for testing the hypothesis that the effect of prenatal TP is permanent. The difference might not have been compelling in this test as reflected in the authors’ reticent suggestion that the effect “*appears* to be permanent” (italics added). Thus, the suppression of lordosis was convincingly demonstrated in animals with a male brain and a penis (hermaphrodites and males), but less convincingly in animals with a male brain but no penis (unmodified females). These findings make the separation of the “neural” among “the tissues” mediating lordosis behavior difficult.

The animals had been prepared “for a study of the structural changes in the gonads, genital tract, and external genitalia” (Phoenix et al., 1959, p 370, footnote 3), but effects were reported only for the external genitalia. On the basis of the absence of a penis, it was suggested that the neural tissues mediating mating behavior rather than the genital anatomy had been organized, but the penis is not part of the tissues mediating lordosis behavior. By contrast, the flank-perineum skin area, which had been stimulated manually, is among those tissues. Interestingly, Kun (1937) had reported that the skin is a target for estrogen, and there was an extensive literature on the effects of gonadal hormones on the skin, including sex differences in the response to estrogen and androgen (Burrows, 1949; Rothman, 1954). Some years later, it was confirmed that the skin area of the female rat that the male stimulates during copulation is enlarged by estrogen (Komisaruk et al., 1972; Kow and Pfaff, 1973), and that anesthetizing that skin area markedly decreases the display of lordosis (Hansen et al., 1980).

### The problems with mounting in response to EB plus P

The suggestion that mounting increased in prenatally TP-treated animals without hormone treatment was supported by the findings in the hermaphrodites and the males but not by the findings in the unmodified females. However, the suggestion, that “the capacity to display male-like mounting was not suppressed” in response to treatment with EB plus P was supported by the results in the unmodified females, but was inconsistent with the results in the hermaphrodites and males in the first test. Hence, the crucial group of animals with a male brain but without a penis, the unmodified females, was not masculinized (i.e., insensitive to estrogen) in this test. By contrast, the hermaphrodites were, but they, of course, also had a penis. Once again, the male brain–penis dissociation was not clearly supported by the data.

The results on mounting in the retests are intriguing. Thus, the unmodified females mounted twice as much and the three hermaphrodites mounted almost five times as much as the controls in the final test. These results are inconsistent with the hypothesis that animals with a male brain are insensitive to estrogen. Although the males were not retested, a subsequent study confirmed that EB plus P-treated hermaphrodites mount more than control females, but that similarly treated males do not (Goy et al., 1964). Female guinea pigs treated prenatally with TP are therefore not comparable to males in this respect.

### The problem with mounting in response to TP

The finding that the hermaphrodites and the males mounted more than the control females in response to TP treatment in adulthood supported the suggestion that the tissues mediating mating behavior of the hermaphrodites were organized in a manner similar to the tissues of males. However, the absence of unmodified females in this experiment makes it impossible to relate this effect to an anatomy that is separable from the genital anatomy. In subsequently reviewing the results on TP-induced mounting, Young (1961) concluded that the effects were permanent, but this possibility had not been tested.

### The failed liberation of the neural tissues mediating mating behavior

Although Phoenix et al. (1959) reported only the presence or absence of a penis, it had already been shown that prenatally administered TP exerts a dose-dependent, continuous effect on the internal as well as the external genital organs of female rats (Green and Ivy, 1937; Greene et al., 1939). These anatomical effects were subsequently replicated in the guinea pig (Goy et al., 1964), and one wonders therefore whether unmodified females are actually internally unmodified, and, thus, whether the brain of a female guinea pig can be modified by prenatal TP treatment while the non-neural parts are not. And one also wonders why there were no unmodified females in the study on the effect of treatment TP on mounting (Phoenix et al., 1959) and in the study of the effect of EB plus P treatment on lordosis (Goy et al., 1964). The absence of this group makes it difficult to separate the neural from the genital parts among the tissues mediating mating behavior.

## Estrogen, Sex and Internal Secretions, and the Nobel Prize

The encyclopedic *Sex and Internal Secretions* (Royal Society of Medicine, 1962) was a “monumental, indispensable work, covering all aspects of the subjects including sexual behaviour.” In the 1939 edition, Gustavson (1939) discussed the synergistic effect of estrogen and androgen in the fibromuscular layer of the seminal vesicles that Freud (1933) had reported and that Steinach et al. (1936) had extended to the sexual behavior of male rats. In the 1961 edition, Price and Williams-Ashman (1961) discussed these estrogen–androgen interactions again, and Villee (1961) outlined the metabolism of testosterone to estrogens. Young (1961) spent 4 years preparing the 1961 edition and “… read every word in the manuscripts submitted by the contributors …” (Gerall, 2009). It is paradoxical therefore that he did not discuss the role of the conversion of testosterone to estrogen for the display of sexual behavior that Steinach et al. (1936) had reported in his own account of “The Hormones and Mating Behavior” (Young, 1961).

The belief that males are insensitive to estrogen had become so established that the rediscovery of the potent effect of estrogen on male sexual behavior came as a surprise: “Normally androgen is much more potent than estrogen in its ability to maintain or restore masculine sexual performance … these animals must have been extremely sensitive to the activational influence of such comparatively small quantities of estradiol” (Baum, 1972).

In reviewing the article by Steinach et al. (1936) in 1938, the Nobel Prize Committee had pointed out the potency of estrogen in stimulating sexual behavior in male rats: “Steinach’s studies of the sensory control of testicular function led him to examine the mechanism of hormone action. While EB has a strong effect on the blood flow in the brain, androgens must be given in high dosages to be effective. Steinach et al. (1936) explain this difference by the conversion of androgens to estrogen, which is necessary for an effect of androgen on the brain, as evidenced by the display of sexual behavior … a very low dose of EB reduced the dose of androgen to 1/(10-42.5) of what was otherwise needed” (Liljestrand, 1938), Zondek (1934a,b; Zondek and von Euler, 1934). who had discovered that androgens are converted into estrogens in males, was also nominated for the Nobel Prize in 1938.

It had also been shown that treatment with EB decreases the threshold for induction of ejaculation by cutaneous electrical stimulation 8-fold for testosterone and 40-fold for TP in castrated rats (Kun and Peczenik, 1937), and similar estrogen–testosterone interactions on ejaculation had been described in a patient (Foss, 1937, 1939). These studies were reviewed in the overview (Burrows, 1949) to which Phoenix et al. (1959) and Young (1961) referred.

Thus, there were some exceptions to the organization-activation framework at the time, and a few more have since been added.

## The Elusive Search for the Male Brain

The results on lordosis in the hermaphroditic guinea pigs suggested, of course, a “role of the developing testis in differentiation of the neural tissues mediating mating behavior” in the male (Grady et al., 1965). Its short gestation period made the rat the model of choice for testing this hypothesis; the testes can be removed postnatally, and prenatal castration would be required but difficult in the guinea pig.

Accordingly, lordosis was readily induced in rats castrated before 10 d of age, but treatment with as much as 165 μg of EB plus P had essentially no effect if the rats were castrated at 50 d of age (Grady et al., 1965). This dose of EB is 30 times higher than the threshold dose used by Kun (1934) to induce lordosis in adult castrated male rats.

**Figure 2.**
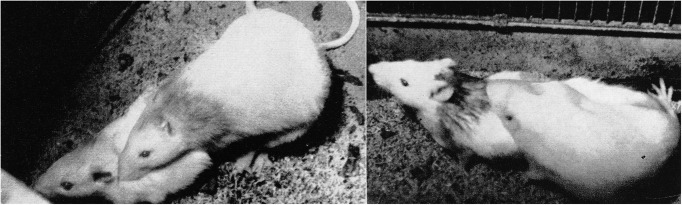
Mounting and lordosis in a castrated male rat (stained neck) treated with 50 μg of testosterone propionate and 80 μg of dienestrol diacetate. Reproduced from Hohlweg et al. (1962) with permission.

However, Hohlweg et al. (1962) had replicated the findings of Kun (1934) that estrogen induces bisexual behavior in male rats (Fig. 2), and research over the next decade yielded some other inconsistencies in the search for the male brain (Beach, 1971). And even today, when many sexually dimorphic brain areas have been discovered, it has proven difficult to relate any of these causally to a sex difference in sexual behavior (Arnold and Breedlove, 1985; Balthazart et al., 1996; de Vries and Södersten, 2009). A partial explanation of these discrepancies has been suggested by the results of experiments on the sex difference in lordosis in rats and guinea pigs.

### Ovarian control of lordosis

Young (1961) had performed elegant, compelling experiments showing that the ovaries control the display of sexual receptivity in rats and guinea pigs. However, most experiments on this topic have used methods of hormone administration that are unrelated to the normal pattern of hormone secretion by the ovaries (e.g., Jones et al., 2013). As pointed out 91 years ago, this may be misguided:

“It seems to me also that the desire to replace an endocrine gland by the injection of an extract from the respective organ arises from a too purely morphological attitude. *In reality it will never be possible to accomplish such a substitution until we are able to imitate quantitatively the rate and rhythm of the secretory action of the gland*” (Lipschütz, 1924).

However, the substitution has been accomplished in rats and guinea pigs, and the results are relevant to Steinach’s concept of organization (i.e., that the presence of the gonads of one sex in an individual of the other sex results in “psychosexual transformation” of the sexual behavior of that individual into the sexual behavior of the individual of the other sex).

Thus, a study using transplantation (i.e., the method Steinach used early on) showed that the presence of ovaries during development facilitates the display of lordosis by neonatally gonadectomized female and male rats (Gerall et al., 1973). A new study confirmed this effect (Brock et al., 2011) as did an old one (Södersten, 1976), which, in addition, showed that the presence of the ovaries eliminates the inhibitory effect of neonatal TP treatment on the display of lordosis in adulthood by female rats.

**Figure 3.**
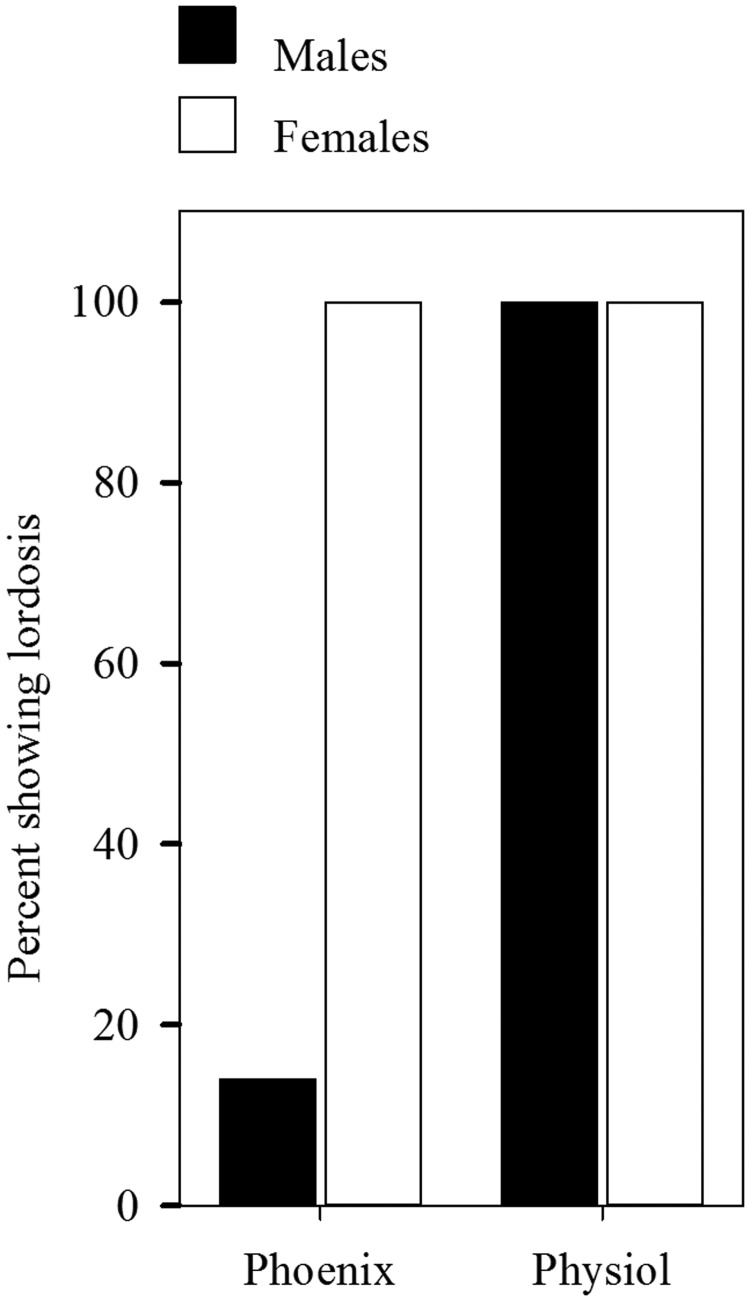
Replication of the marked sex difference in lordosis in guinea pigs treated with 10 μg of estradiol benzoate and 0.5 mg of progesterone (Phoenix), and elimination of the difference by treatment with two injections of 2 μg of estradiol and 0.5 mg of progesterone (Physiol). Reproduced from Olster and Blaustein (1990) with permission.

These results generated the hypothesis that imitating the secretions of the ovaries by the injection of estradiol and P in gonadectomized rats might abolish the sex difference in the display of lordosis. A series of experiments on rats verified this hypothesis (Södersten et al., 1983; Olster and Blaustein, 1988; reviewed in detail by Södersten, 2012). The hypothesis has also been verified in the guinea pig. Thus, the sex difference in lordosis that Phoenix et al. (1959) reported was first replicated and then eliminated by treating the animals with estradiol in a manner that more likely mimics the physiological pattern of estrogen secretion by the ovary (Fig. 3; Olster and Blaustein, 1990).

These results show that if we “*imitate quantitatively the rate and rhythm of the secretory action of the* [*ovary*],” the sex difference in sexual receptivity is eliminated. Studies in which sex differences have been reported made no attempt at such an imitation (Becker et al., 2005).

## Concluding Remarks

It appears that Phoenix et al. (1959) said the right thing at the right time. By contrast, Steinach said the right thing at the wrong time; his ideas were ahead of his time and therefore “… their final test was delayed for half a century …” (Beach, 1981). Thus, it took 37 years before the effect of combined estrogen-androgen treatment on the sexual behavior of the male rat (Steinach et al., 1936) was rediscovered (Baum and Vreeburg, 1973; Larsson et al., 1973; Feder et al., 1974), and another 39 years before it was realized that Steinach had reported the effect 76 years earlier (Södersten, 2012). It seems likely that differences in theoretical perspectives, at least in part, explain why Steinach’s ideas hibernated for such a long time.

While Phoenix et al. (1959) were undoubtedly right in suggesting that prenatal androgen organizes the brain, that effect, however, may not be permanent. Interestingly, it was recently pointed out that “The original formulation of the Organizational Hypothesis didn't claim that a system once organized could not be reorganized” (Wallen, 2009). This concept of reorganization is conspicuously similar to Steinach’s concept of development (Entwicklung), and so, in the end, the work of Steinach and Young may come together.

However, it is not surprising that the impressive research carried out by Young’s group over many years and culminating in the article by Phoenix et al. (1959) has exerted such a strong influence. The work illustrates the strength of a conceptual framework in stimulating research, as shown by the impressive work on the role of perinatal androgen in the development of the preoptic area of the brain (Nugent et al., 2015). The importance of the preoptic area in male sexual behavior has long been recognized, although it is also long known that mounting and ejaculation can be shown by male rats in which this part of the brain has been removed after the neonatal period when the brain has been organized into a male brain (Twiggs et al., 1978). However, the fact that there are some exceptions to the organizational hypothesis does not detract from its usefulness in both teaching and research.

## Synthesis

The decision was a result of the Reviewing Editor Jeffrey Blaustein and the peer reviewers coming together and discussing their recommendations until a consensus was reached. A fact-based synthesis statement explaining their decision and outlining what is needed to prepare a revision is listed below. The following reviewers agreed to reveal their identity: Raul Paredes.

The reviewers are now very positive regarding this manuscript. There are now only two very, very minor issues that need to be corrected: LINE 8: Abstract 1930s, not 1930ies; Line 12: 1950s, etc. Line 32 and 44 says estrogen's effects were rediscovered in 1973, but line 41 dates it to 1970. Choose one, or lump them together and say early 1970s.
